# The clinical characteristics of older people with chronic multiple-site joint pains and their utilisation of therapeutic interventions: data from a prospective cohort study

**DOI:** 10.1186/s12891-016-1049-0

**Published:** 2016-04-30

**Authors:** Rafi Raja, Bright Dube, Elizabeth M. A. Hensor, Sarah F. Hogg, Philip G. Conaghan, Sarah R. Kingsbury

**Affiliations:** Leeds Institute of Rheumatic and Musculoskeletal Medicine, Chapel Allerton Hospital, University of Leeds and NIHR Leeds Musculoskeletal Biomedical Research Unit, Chapeltown Rd, Leeds, LS7 4SA UK

**Keywords:** Joint pain, Multiple site, Osteoarthritis, Back pain, Soft tissue disorders, Therapeutics

## Abstract

**Background:**

Chronic multiple-site joint pain (MSJP) is common in older people and associated with poor outcomes, yet under-researched. Our aim was to detail the clinical characteristics of people with MSJP and their utilisation of therapies.

**Methods:**

MSJP was defined as pain in at least one large joint and one other joint for >6 weeks in the last three months. A mixed community, primary and secondary care cohort of people >50 years old underwent detailed history and examination by a single clinician. Treatment utilisation was recorded comprehensively.

**Results:**

201 adults were recruited, 82 % women, mean age 63, BMI 31 kg/m^2^. Median number of painful joints per patient was 6 (IQR 4–9; range 2–17); most common painful sites were knee (84 %), lower back (62 %) and shoulder (47 %). 194/201 (96 %) had an osteoarthritis (OA) diagnosis, 155/194 (80 %) also had soft tissue pathology and 72 % had back problems. 85 % had OA at multiple sites. Upper and lower limb weakness was common (90 and 77 % respectively). Lower limb weakness was significantly associated with obesity. Only 26 % had received written information about their joints. Though 79 % had attended physiotherapy, the majority (93 %) had muscle weakness. Only 36 % of overweight participants had accessed weight-loss support. Half of those with foot pain had seen a podiatrist or used appliances. Multiple concurrent pharmacological therapies were used by 47 %.

**Conclusion:**

MSJP represents a combination of OA, back pain and soft tissue disorders; muscle weakness is extremely common. Therapies appear underutilised in people with MJSP. Identifying the reasons for this should guide effective intervention research.

**Electronic supplementary material:**

The online version of this article (doi:10.1186/s12891-016-1049-0) contains supplementary material, which is available to authorized users.

## Background

Musculoskeletal (MSK) problems are now the second most significant cause of disability worldwide, with low back pain remaining the leading specific cause of years lived with disability, and osteoarthritis (OA) significantly rising in importance [1]. Large epidemiological studies have reported that chronic multiple-site joint pain (MSJP) is more common than single joint problems in older adults and is associated with poor outcomes [2, 3]. Increased numbers of painful joints is related to poor physical function [3, 4] and increased work disability [5].

Despite the frequent prevalence of MSJP and the associated poor outcomes, the characteristics of MSJP have not been well described or researched. Of note, there have been extremely few therapeutic trials in this area. The majority of MSK pain trials have involved selecting a single predominantly painful joint, whilst guidelines have focused on individual disease areas such as OA or back pain [6, 7]. The effectiveness of currently available therapies in people with MSJP is therefore not known. A recent survey of the approach of UK general practitioners (GPs) on pharmacological management of MSJP found that most did aim to treat multiple-site pains concurrently, using the same therapies for all sites irrespective of diagnosis [8].

Although clinicians have long recognised MSJP, the lack of characterisation and understanding of this condition, and the lack of management strategies, in the context of a rapidly ageing and increasingly obese society, makes this an increasingly important area for further research. The aim of this study was therefore to examine the detailed clinical characteristics of people with MSJP and their utilisation of therapeutic interventions.

## Methods

### Study population and eligibility criteria

Prospective participants were identified through the following sources: referral by general practitioners from primary care services; referral by physiotherapists from musculoskeletal services; identification by clinicians within secondary and tertiary care rheumatology, musculoskeletal and orthopaedic clinics; patient public involvement organisations in West Yorkshire. Patients were screened via a telephone interview and those meeting the inclusion criteria were recruited.

The inclusion criteria were patients aged 50 years and above, having pain in at least one large joint and one other joint for more than six weeks within the last three months, and capable of understanding and signing an informed consent form. The definition of a large joint area in this study included the spine, shoulders, elbows, hips, knees and ankles. Exclusion criteria included i) previous diagnosis of a primary inflammatory arthritis including rheumatoid arthritis, gout, polymyalgia rheumatica or connective tissue disease, ii) previous clinician-diagnosed fibromyalgia, iii) a chronic medical condition requiring long term use of oral corticosteroids or immunosuppressants and iv) unable or unwilling to give informed consent.

### Ethics, consent and permissions

Ethical approval was received from the Yorkshire and the Humber (Leeds Central) Ethics Committee (Ref: 12/YH/0345) and all participants gave written informed consent.

### Design

The Leeds MSJP study involves observational, longitudinal evaluation of this under-researched group; here we present the baseline cross-sectional data. Participants completed a series of questionnaires and underwent a detailed history and clinical examination of painful joints by a single rheumatologist to document medical history and musculoskeletal diagnoses. A patient and public involvement group representative was involved in the development of the study and design of the participant case report form.

Information on age, gender, ethnicity, smoking status, alcohol consumption, employment history and medical co-morbidities, including joint related co-morbidities (duration of joint pain, previous joint surgery and family history), and comprehensive therapy use (current and previous pharmacological therapies, including over-the-counter (OTC), and local therapies) were recorded (see Additional file 1). Clinical diagnoses for the painful joints were based on established diagnostic criteria (Additional file 1). Where diagnostic criteria were not available, the clinician’s judgement was used. Where previous radiographic information was available, this was used to support the clinical diagnosis. Upper limb strength was measured using a Jamar hand dynamometer (Sammons Preston Rolyan, Bolingbrook, IL, USA). Three attempts were recorded (measured in pounds) for each hand, and the three trials then averaged to create scores for analysis. For lower limb muscle strength, the Medical Research Council (MRC) scale was used. The MRC scale is an established test for muscle grading on a 0 to 5 scale which has been previously shown to be reliable for assessing lower limb weakness [9] (Additional file 1).

Participants completed a set of questionnaires that asked about their joint pain and function over the past week. Joint pain was also assessed using a manikin that included the neck, upper back, lower back, shoulders, elbows, wrists, hands or fingers, thumbs, hips, groin, knees, ankles, feet and ball of feet or toes. 11-point numerical rating scales were used to assess overall pain severity from all joints and pain from the most painful joint.

### Statistical analysis

Statistical analysis was carried out with Stata 13.1 software (StataCorp LP, College Station, TX, USA) [10] and R version 3.1.1 [11].

Descriptive statistics were used to describe the main characteristics of the study population and are presented as arithmetic mean (S.D) or medians where appropriate for continuous variables and frequency and percentages for categorical variables. The prevalence of pain at each site was evaluated and correlations between reported pain at each site compared with other sites using phi correlation coefficients for binary data. These associations between pain at pairs of sites were also explored using logistic models and findings summarised using odds ratios (ORs) and 95 % confidence intervals having adjusted for age and sex. Muscle weakness was split into upper limb (based on hand grip measurements) and lower limb (using quadriceps strength measurements). For upper limb strength, the average of 3 attempts from the dynamometer readings was used, these were then converted to kilograms and the determination of whether an individual’s upper limb was weak was adjusted based on their gender and BMI. For males the cut offs were: (obese men < 40 kg; overweight men <39 kg and normal men 37 kg) while in female the cut off was uniformly 21 kg for one to be considered to have weakness in that limb [12]. For lower limb weakness any score less than 5 on the MRC scale was considered as weakness for that limb. The MRC scale grades weakness from 0 to 5 with zero being no movement observed and five representing the muscle fully contracting under resistance (Additional file 1). Associations between site of muscle weakness (upper limb weakness vs lower limb weakness) and associations between limb pain and muscle weakness (upper limb pain vs upper limb weakness and lower limb weakness vs lower limb pain), and also associations between muscle weakness and specific joint pain (for example lower limb weakness vs shoulder pain) were evaluated using chi-square (*Χ*^2^) tests or Fisher’s exact test where appropriate. Differences were considered significant at *P* < 0.05.

## Results

Among 424 people who were approached to participate in the study, 210 people were screened. Three failed screening (fibromyalgia *n* = 2, chronic illness on long term corticosteroid therapy *n* = 1). Six participants with pain limited to one large joint at the time of the baseline assessment were not included in this analysis, leaving 201 participants. The mean ± SD age of the cohort was 63 ± 8.77 years (range 50–88) with 82 % of the cohort being female. The mean BMI ± SD was 31 ± 6.42 kg/m^2^. The mean duration of symptoms was 13.7 years (range 1–50). Table 1 shows the demographics and baseline characteristics of the participants.

**Table 1:** Demographics and baseline characteristics

Characteristic	*N* (%)
Age, years, mean ± SD (range)	63 ± 8.77 (50–88)
Female	164 (82)
BMI, kg/m^2^, mean ± SD	31.0 ± 6.42
Ethnicity	
Caucasian	193 (96)
African/Caribbean	5 (3)
Asian	3 (1)
Smoking status	
Current smoker	16 (8)
Previous smoker	81 (40)
Pack years, mean (range)	20 (1–75)
Alcohol consumption	
Units per week, mean (range)	4 (0–38)
Employment history	
Employed	49 (24)
Self-employed	14 (7)
Retired	110 (55)
Unemployed	28 (14)
Job activity (current or previous)	
Heavy manual	94/192 (49)
Repetitive use of hands	21/192 (11)
Prolonged key boarding or typing	46/192 (24)
Prolonged standing	26/192 (14)
Median number of painful joints (IQR)	
Overall in all participants	6 (4–9)
Current/previous smokers	7 (5–9)
Never smoked	6 (4–8)
Heavy manual job	7 (4–9)
Repetitive use of hands	7 (6–10)
Prolonged keyboarding/typing	6 (5–8)
Prolonged standing	6 (5–8)
Comorbidities	
Cardiovascular disease (including hypertension, ischaemic heart disease)	94 (47)
Pulmonary condition (including asthma, emphysema, chronic bronchitis)	44 (22)
Gastro-intestinal disease (including gastro-oesophageal reflux, peptic ulcer)	108 (54)
Diabetes	20 (10)
Depression	63 (31)
Medical history	
Joint replacement or fusion	57 (28)
Joint related soft tissue repair	53 (26)
Non-orthopaedic surgery	160 (80)
Family history of OA	135 (67)

*BMI* body mass index, *IQR* interquartile range

### Examination findings

A total of 1342 painful joints were reported by participants with the median number of painful joints per person being six (inter-quartile range 4–9, range 2–17). Peripheral joint pain was more common (82 %, 1107/1342) than axial joint pain (18 %, 235/1342). The most commonly involved painful joints by participant were the knee (84 %, 168/201), lower back (62 %, 125/201) and shoulder (47 %, 95/201), while the least commonly painful was the foot excluding the toes (12 %, 25/201). The joint with the highest proportion of bilateral pain for that particular joint was the hand/finger with 90 % of all the participants with involvement in that joint having the condition affecting both sides. This was followed by the thumb (71 %) and knee (67 %).

Of the painful joints, 51 % (688/1342) were diagnosed with OA, 30 % (408/1342) as having soft tissue pathology, 18 % with axial pain and 1 % non-specific joint pain. Most participants (96 %, 194/201) had at least one joint with an OA diagnosis, 81 % (162/201) had soft tissue pathology and 77 % (155/194) had both OA and soft tissue pathology. The prevalence of soft tissue pathology was as common as OA in the upper limbs whereas OA was more common in the lower limbs (Table 2).

**Table 2:** Symptomatic joint and diagnosis

	*N* (%) by region	(%) of total number of joints
Upper limb		
Osteoarthritis	277/520 (53)	20
Soft tissue pathology	242/520 (47)	18
Lower limb		
Osteoarthritis	411/588 (70)	31
Soft tissue pathology	166/588 (28)	12
Other (including referred pain)	11/588 (2)	1
Axial		
Mechanical	219/235 (93)	16
Mechanical with radiculopathy	16/235 (7)	2

The most common joint-specific diagnosis was knee OA (20.8 %, 279/1342) followed by hand OA (11.2 %, 150/1342), trochanteric bursitis (9.6 %, 129/1342), subacromial impingement syndrome (9.5 %, 128/1342), thumb OA (9.2 %, 124/1342) and mechanical low back pain (8.3 %, 112/1342). OA accounted for 99 % of knee pain, 92 % of hand pain, 86 % of foot/ball-of-foot/toe pain and 74 % of groin pain. 99 % of “hip” pain was due to trochanteric bursitis and 96 % of shoulder pain was due to subacromial impingement syndrome.

**Fig. 1:**
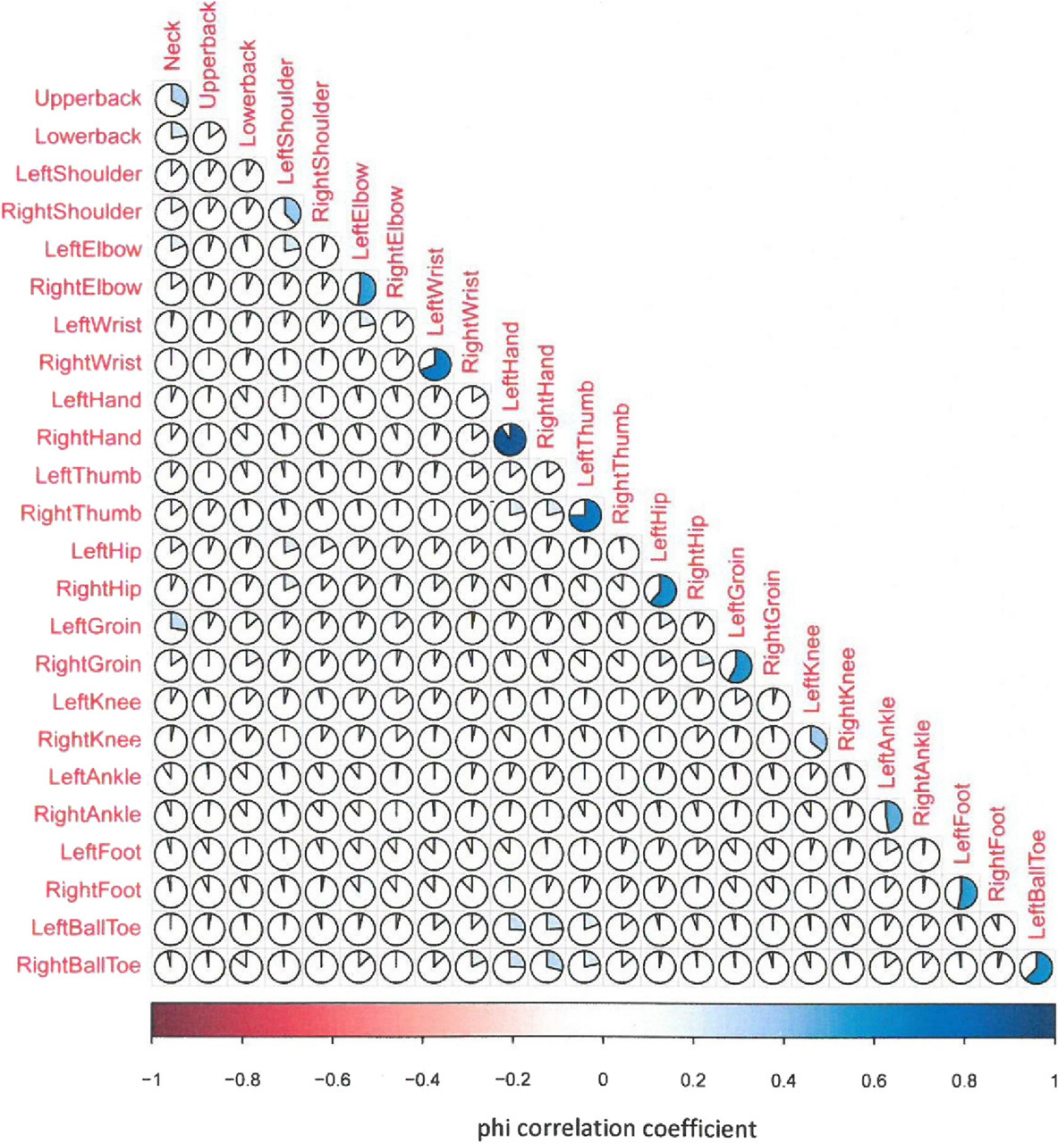
Association between painful joints. Clockwise shading represents positive correlation and anti-clockwise shading represents negative correlation

Figure 1 shows the correlations of pain at each of the 25 anatomical sites. Pain was reported most frequently in the right knee (73.6 %), left knee (66.2 %) and lower back (62.1 %), and least often in the left ankle (8.0 %) and right foot (8.5 %). Higher correlations were observed for pain at bilateral joints (correlation for right hand and left hand 0.91; left and right thumbs 0.75) and also anatomically adjacent joints (shoulder with ipsilateral elbow 0.22 and groin and ipsilateral hip 0.21). Exploratory analyses using adjusted logistic regression models revealed similar results but with wide confidence intervals. The strongest associations were for pain at corresponding sites bilaterally (ORs 706 for right and left hand, 95.2 for right and left wrists, 56.2 for right and left thumb). Higher ORs were also observed for pain at adjacent anatomical sites in the upper limb (shoulder with ipsilateral elbow, 4.7), and lower limb (right groin with ipsilateral hip, 4.3) (Additional file 2: Table S1).

#### Muscle weakness

Four participants were unable to perform the handgrip strength test due to hand pain, whilst 21 participants were unable to complete the quadriceps strength test due to lower limb pain. Muscle weakness was very common, affecting the upper limb in 90 % (178/197), lower limb in 77 % (139/180), and both locations in 74 % (131/178). There was a statistically significant association between lower limb weakness and upper limb weakness (*Χ*^2^ = 20.21, *p* < 0.001). 90 % of participants with upper limb pain had upper limb muscle weakness (149/165) and 78 % with lower limb pain had lower limb weakness (134/172). A statistically significant association was also demonstrated between having lower limb weakness and having shoulder pain (*Χ*^2^ = 8.39, *p* = 0.004), with 87 % of participants with shoulder pain having lower limb weakness compared to 69 % of participants that had no reported shoulder pain. In participants classified as obese (BMI > 30), 86 % of these reported lower limb weakness compared to 69 % of those not classified as obese (*Χ*^2^ = 7.11, *p* = 0.008).

### Therapy

#### Information

Provision of written information about the participant’s joint problem was infrequent, with only 26 % (53/201) recalling receiving such information or being referred to other resources (e.g. internet website) following a GP or specialist consultation.

#### Non-pharmacological treatment use

Non-pharmacological therapy use was infrequent. 173 participants were classed as being overweight, of which 36 % (62/173) recalled receiving weight loss treatment (advice, written information, weight loss programme or dietician input). The majority (79 %, 159/201) had attended physiotherapy, 62 % (99/159) of these reported continuing their exercises. Only half (51 %, 32/63) of those with foot pain had used a podiatry service or a shoe appliance. The most common reason for using specific non-pharmacological therapies were: physiotherapy for knee OA (40 %, 81/201) and mechanical neck/back pain (39 %, 79/201), exercise for knee OA (33 %, 67/201), transcutaneous electrical nerve stimulation (TENS) for back pain (16 %, 33/201), acupuncture for back pain (21 %, 43/201), occupational therapy for hand/thumb OA (10 %, 20/201), appliances for hand/thumb OA (17 %, 34/201) and surgery for knee OA (20 %, 40/201).

#### Current pharmacological treatment use

Analgesia use was self-reported by 95 % of participants. Of these, 71 % were using one or more prescription medications, 74 % were using over-the-counter (OTC) medication and 44 % were using both prescription and OTC medications. Paracetamol (62 %) was the most commonly used medication, with 62 % of paracetamol users reporting this as OTC use. Opioids were the second most common medication (51 %) and were mainly reported as prescription medication (81 % for co-codamol and 100 % other opioids) (Table 3). Oral co-codamol (a fixed combination agent including paracetamol and codeine) was the most commonly used opioid medication, although other oral opioid (codeine phosphate, tramadol, oral morphine) use was also reported. Transdermal opioid use was low (buprenorphine patch and fentanyl patch). Most participants on opioid therapy used a single opioid (77 %, 79/103) rather than combination oral opioid therapies (8 %, 8/103). NSAIDs were used in 38 % (77/201), with the majority using a non-selective NSAID (*n* = 71). Almost half of NSAID users reported them as an OTC medication (44 %) Gastro-protective agent (GPA) use among all non-selective NSAID or COX-2 inhibitor users was 48 % (34/71) and 67 % (4/6) respectively. In participants taking OTC NSAIDs, only 35 % of these used a GPA compared to 58 % of those taking prescription NSAIDs. Nutraceutical use was limited with only 6 % reporting use of glucosamine/chondroitin (Table 3).

**Table 3:** Overall pattern of current and previous medication use

	Paracetamol	NSAID	Cox2 inhibitor	Oral co-codamol	Other oral opioid^a^	Transdermal opioid	Glucosamine/Chondroitin	Hydroxychloroquine
Current drug use	124 (62)	71 (35)	6 (3)	58 (29)	47 (23)	14 (7)	12 (6)	4 (2)
Prescription drug	47 (38)	40 (56)	6 (100)	47 (81)	47 (100)	14 (100)	0 (0)	4 (100)
Reason for use								
For most painful joint	68 (55)	46 (65)	3 (50)	36 (62)	22 (47)	2 (14)	1 (12)	2 (50)
For overall pain	56 (45)	25 (35)	3 (50)	22 (38)	25 (53)	12 (86)	11 (88)	2 (50)
Drug experience								
Effective	50 (40)	49 (69)	3 (50)	32 (55)	29 (62)	6 (43)	7 (58)	3 (75)
Not effective	74 (60)	22 (31)	3 (50)	26 (45)	18 (38)	8 (57)	5 (42)	1 (25)
Side effects	3 (2)	12 (17)	6 (100)	24 (41)	24 (51)	3 (21)	0 (0)	0 (0)
Frequency of use								
Regular use	28 (23)	18 (25)	5 (83)	19 (33)	22 (47)	12 (86)	11 (88)	4 (100)
Only when in pain	96 (77)	53 (75)	1 (17)	39 (67)	25 (53)	2 (14)	1 (12)	0 (0)
Previous drug use	52 (26)	119 (60)	4 (2)	87 (43)	53 (26)	9 (4)	70 (35)	5 (3)
Reason stopped^b^								
Side effects	1 (2)	78 (66)	4 (100)	52 (60)	43 (81)	4 (44)	4 (5)	4 (80)
Inefficacy	42 (81)	32 (26)	0 (0)	20 (23)	6 (11)	3 (33)	58 (83)	1 (20)
Loss of efficacy	9 (17)	7 (5)	0 (0)	7 (7)	1 (2)	0 (0)	3 (4)	0 (0)
Doesn’t like taking pills	0 (0)	1 (1)	0 (0)	5 (5)	1 (2)	2 (22)	4 (5)	0 (0)
Pain not severe enough	0 (0)	4 (3)	0 (0)	5 (5)	2 (0)	1 (11)	2 (3)	0 (0)

Values shown are number of participants (%)

^a^Other oral opioid includes codeine phosphate, tramadol and oral morphine

^b^Some participants had more than one reason for stopping medication

Combination oral analgesia use was common (59 %, 113/191) and is outlined in Table 4. The most common local pharmacological therapies were topical NSAID for knee OA (43 %, 87/201) and intra-articular corticosteroid injection for subacromial impingement syndrome (24 %, 49/201) and knee OA (24 %, 49/201).

**Table 4:** Analgesic combinations in 191 participants ^a^

Type of treatment	Number of patients (%)
Monotherapy	78 (41)
Paracetamol	36 (19)
NSAID	12 (6)
Opioids	38 (20)
Nutraceuticals	0 (0)
Combination therapy	113 (59)
Paracetamol	
with NSAID	31 (16)
with opioids	30 (16)
with nutraceuticals	4 (2)
with NSAID and opioids	15 (8)
with NSAID and nutraceuticals	3 (2)
with opioids and nutraceuticals	3 (2)
NSAID	
with opioids	12 (6)
with nutraceuticals	1 (1)
with opioids and nutraceuticals	1 (1)
Opioids	
with nutraceuticals	2 (1)
Nutraceuticals	
with NSAID and opioids	1 (1)
with all drugs	2 (1)

*NSAID* nonsteroidal anti-inflammatory drugs

^a^ 10 participants did not take any medication

Participants using non-selective NSAIDs or oral co-codamol tended to use the analgesia for the most painful joint rather than for overall pain. Most participants did not use oral analgesia regularly, instead reporting to use it only when in pain. In general efficacy was lowest among participants that reported use of paracetamol (40 %, 50/124) and highest for NSAID (69 %, 49/71) and other oral opioid users (62 %, 29/47). NSAID and co-codamol users who found their medication to be effective were significantly more likely to be using it for their most painful joint (36/49, 73 % and 25/32, 78 % respectively) than for all of their joints (13/49, 27 %, *Χ*^2^ = 5.22, *p* = 0.022; 7/32, 22 %, *Χ*^2^ = 7.82, *p* = 0.005). For paracetamol users and opioid users effectiveness was equally as likely in participants reporting use for their most painful joint or for overall pain. Side effects were least common among paracetamol users and most common in those using oral opioid therapy. The overall pattern of current pharmacological therapy use is described in Table 3. The most common reason for stopping NSAID and all opioid (oral and transdermal) medication was side effects whereas inefficacy was the most common reason for stopping paracetamol and glucosamine/chondroitin (Table 4).

## Discussion

This is the first comprehensive study to examine the detailed clinical characteristics of whole-body MSJP and peoples’ utilisation of therapeutic interventions. This study confirms that MSJP is a heterogeneous condition comprised of a range of musculoskeletal pathologies. It can be conceptualised as different combinations of peripheral joint OA, soft tissue disorders and mechanical back pain.

In this study we included participants on the basis of at least one large joint with pain. Previous studies have specified knee OA as the index joint whilst others have reported patients with multiple painful joints only if they meet the ACR criteria for fibromyalgia; such studies therefore do not necessarily reflect a true representation of mechanical MSJP patients [13–15]. Many previous MSJP studies, whether postal questionnaire or interview based, have reported the number of painful joints without characterisation of the involved joints [16]. Our study included detailed clinical diagnosis of all involved joints, both upper and lower limbs as well as the axial joints. We found that nearly all participants had at least one joint with an OA diagnosis, with most also having soft tissue pathology in at least one joint. A previous study which characterised upper limb joint pain reported that specific soft-tissue disorders were common in the upper limb [17]. In line with this, we found that soft-tissue disorders were common in the setting of MSJP especially in the upper limb compared to the lower limbs. This study highlights the heterogeneity of MSJP and demonstrates that a combination of OA, back pain and soft tissue disorders is common in this phenotypic setting.

Association between reported painful sites was strongest for contralateral and adjacent sites, which may be attributed to shared risk factors. Our findings are similar to those from a 12,400 patient questionnaire study which found similar associations (ORs) for self-reported pain at 10 anatomical sites [18].

Muscle weakness captured as a component of MSJP in previous studies has been limited to the upper limbs. Andersson et al. observed that individuals with generalised pain were more likely to have reduced hand muscle strength compared to regional pain [19]. We recorded both upper and lower limb muscle strength and found that both were common in the setting of MSJP. Obesity and shoulder pain showed a statistically significant association with lower limb muscle weakness, whilst the majority (87 %) of participants with shoulder pain had lower limb weakness. These results suggest that obesity and/or lower limb muscle weakness may contribute to upper limb symptoms, perhaps through changes to the use of the upper limbs, as has recently been reported [20]. The longitudinal data from this study may shed more light on this putative biomechanical chain of events.

Previous studies have mainly recorded non-pharmacological management in the setting of knee OA [21, 22]. The majority (79 %) of participants in our study had attended a physiotherapy appointment, with two-thirds of these reportedly continuing with exercises. This is considerably higher than a recent OA study which reported physiotherapy attendance of 46 % [23], but should be interpreted with some caution since we did not capture exact numbers and details of sessions attended or details of adherence. With regards to education, only a quarter of participants recalled ever receiving written information on their joint problem. Although this finding may be influenced by recall bias, nonetheless it suggests that there may be a lack of readily available information (particularly about specific soft tissue disorders) at health practices. This highlights an important area of management that needs further consideration. The majority of participants in this study were overweight/obese (86 %), with only low numbers (36 %) reporting to have ever received any type of weight management therapy. With obesity rates increasing in Western societies, this remains an important issue [24], particularly in light of a recent randomised clinical trial which found that even a modest weight reduction leads to improvement in function in people with knee OA [25].

A recent GP survey revealed a belief that different classes of analgesia have equal efficacy when used across the range of different musculoskeletal pathologies that comprise MSJP [8]. However, research is lacking to examine efficacy across classes of oral analgesia for different musculoskeletal pathologies, especially for opioids, providing limited evidence-base for how to best approach and treat MSJP. Compared to previous studies, this study provides a more detailed description of analgesic use in the setting of MSJP by separately reporting paracetamol, co-codamol, non-selective NSAIDs, cox-2 inhibitors and the different types of opioid (oral and transdermal), as well as examining whether participants used agents for their most painful joint or for overall pain. Although paracetamol was the most commonly used medication in our study, opioid use was frequent with 48 % of participants using at least one opioid. Co-codamol was the most commonly used opioid-containing analgesia, with the large majority prescribed by the GP despite its availability over the counter in the UK. The high use of opioids in our cohort is in line with Grimby et al. who suggested that in their older (>75 years) cohort, ‘light’ opioids were more likely to be used in MSJP than NSAIDs [26]. In contrast our recent study of medication use in people across Europe with OA, found that NSAIDs were the most commonly used medication in this population [27]. The higher use of opioids in the MSJP setting may reflect increased incidence of co-morbidities in this population, which preclude the use of NSAIDs or may reflect physician preference of opioids. In this study, 80 % of participants reported at least one comorbidity related to cardiac, pulmonary or gastrointestinal disease, demonstrating the high prevalence of comorbidities in this population.

Medication efficacy was reportedly higher among those using NSAID or opioid therapy and lowest with paracetamol use. Paracetamol was most commonly stopped due to inefficacy. The lack of efficacy for paracetamol reported by our population is in line with for the very low effect size for paracetamol reported in recent systematic reviews of OA therapies [28, 29]. Notably, for both NSAIDs and co-codamol users, effectiveness was more likely to be reported by participants using the therapy to reduce pain in their most painful joint, compared to those reporting to use the therapy to reduce their overall pain. Most participants did not use oral analgesia on a regular basis, perhaps offering opportunity for optimisation of therapy. Although there is no standard definition for ‘under-utilisation’ in this context, the level of utilisation of therapy which translates into efficacy in such a population is unknown. Further work to establish appropriate levels of therapeutic utilisation and to understand why therapies are currently under-utilised in MSJP is required.

There are limitations to this study. Intra-rater reliability for the different joint diagnoses, which were performed by the single study clinician, was not examined; however the clinician was a fully qualified Consultant Rheumatologist. Although recruitment was from a variety of sources including primary, secondary and tertiary centres, most participants were from the hospital rheumatology clinics. This may have introduced some selection bias, as patients seen in these clinics may have a more complex presentation compared to those managed in primary care. When compared to another Yorkshire community-based postal survey of 16,222 people [2], the median number of painful joints in the present cohort was higher (6 vs. 4). Inclusion of patients with at least one large joint pain may also have introduced some selection bias leading to a cohort mainly with large joint OA, whilst the lack of radiological assessment may have precluded accurate diagnosis of OA in joints not included in ACR criteria e.g. shoulder, elbow and ankle. Whilst we endeavored to capture as many therapies as possible in this study the list was not exhaustive. As such we did not collect information on non-pharmacological therapies such as yoga and Tai Chi, and their current uptake in this group is therefore unknown. There are limitations in using the MRC scale to measure lower limb weakness due to the subjective nature of the test and lack of specificity, which may have resulted in an under-estimation of the true prevalence of lower limb weakness. As with other questionnaire-based studies, findings may also be limited by recall bias.

## Conclusions

In conclusion, MSJP reflects a combination of OA, soft tissue disorders and mechanical back pain. Muscle weakness and obesity are very common within this population. Both systemic and local therapies appear to be underutilised in people with MSJP. Identifying the reasons for this should guide effective intervention research.

## Consent for publication

Not applicable.

## Availability of data and materials

Due to the ongoing collection of longitudinal data for the MultiJoint Study, datasets will be deposited upon study completion. In the meantime, requests for data may be made directly to the authors.

## Additional files

Additional file 1: Methods of data collection. (DOCX 17 kb) Additional file 2: Table S1. Prevalence of pain in the past 6 weeks at 25 anatomical sites and the association with another anatomical pain site. (DOCX 33 kb)

## Abbreviations

ACR: American College of Rheumatology
BMI: body mass index
GP: general practitioner
GPA: gastro-protective agent
MSJP: multiple-site joint pain
MSK: musculoskeletal
NSAID: non-steroidal anti-inflammatory drug
OA: osteoarthritis
OR: odds ratio
OTC: over-the-counter
SD: standard deviation
TENS: transcutaneous electrical nerve stimulation
