# Supplementary material for: The clinical characteristics of older people with chronic multiple-site joint pains and their utilisation of therapeutic interventions: data from a prospective cohort study
Source: BMC Musculoskelet Disord. 2016 Apr 30;17:194. doi: 10.1186/s12891-016-1049-0 (PMC4853864; doi:10.1186/s12891-016-1049-0)
Supplement: Additional file 1: — Methods of data collection. (DOCX 17 kb) [file 12891_2016_1049_MOESM1_ESM.docx]

| **Supplementary Methods**  **Methods of data collection** | |
| --- | --- |
| **Information on therapy use** | **Data collected** |
| Current systemic therapy (oral and transdermal)  Previous systemic therapy (oral and transdermal)  Local (joint specific) therapy  Weight loss treatment  Education on joint pain | Prescription and non-prescription medication including nutraceuticals, reason for using therapy, frequency of use, adverse effects, efficacy of therapy, use of gastro-protective agent (GPA)  Reason for discontinuation  Physiotherapy, self-reported exercise, heat or ice treatment, topical non-steroidal anti-inflammatory drug (NSAID), topical capsaicin, TENS machine, acupuncture, intra-articular corticosteroid injection, occupational therapy, podiatry, wedged insoles, appliances (e.g. wrist splint, knee brace, etc.), joint surgery, joint-related soft tissue surgery  Advice on weight loss, written information, weight loss programme, dietician input  Written information on joint problem, referred to other sources such as an internet website |
| **Muscle strength measurement** | **Methods** |
| Upper limb  Lower limb | Grip strength in both hands measured using a dynamometer, recording the mean of three readings on each side*  Quadriceps muscle strength measured clinically on physical examination using a standardised 5-point muscle strength grading system (0-complete paralysis, 1-flicker of muscle contraction, 2-movement is possible where gravity is excluded, 3-movement is possible against gravity but not if any further resistance is added, 4-movement is possible against gravity and some resistance, 5-normal power)** |
| For grip strength, cut offs to define weakness were based on BMI and gender*** | |
| *Mathiowetz V, Weber K, Volland G, Kashman N. Reliability and validity of grip and pinch strength evaluations. J Hand Surg Am 1984;9(2):222-6  **Talley, N.J., & O’Connor, S. (Eds.). (2010). *Clinical Examination: A Systematic Guide to Physical Diagnosis* (6th ed.). Sydney: Churchill Livingstone Elsevier.  ***Sallinen J, Stenholm S, Rantanen T, Heliovaara M, Sainio P, Koskinen S. Hand-grip strength cut points to screen older persons at risk for mobility limitation. J. Am. Geriatr. Soc. 2010;58(9):1721-6 | |
