# Supplementary material for: The clinical characteristics of older people with chronic multiple-site joint pains and their utilisation of therapeutic interventions: data from a prospective cohort study
Source: BMC Musculoskelet Disord. 2016 Apr 30;17:194. doi: 10.1186/s12891-016-1049-0 (PMC4853864; doi:10.1186/s12891-016-1049-0)
Supplement: Additional file 2: Table S1. — Prevalence of pain in the past 6 weeks at 25 anatomical sites and the association with another anatomical pain site. (DOCX 33 kb) [file 12891_2016_1049_MOESM2_ESM.docx]

| **Table S1. Prevalence of pain in the past 6 weeks at 25 anatomical sites and the association with another anatomical pain site** | | | | | | | | | | | | | | | | | | | | | | | | | | |
| --- | --- | --- | --- | --- | --- | --- | --- | --- | --- | --- | --- | --- | --- | --- | --- | --- | --- | --- | --- | --- | --- | --- | --- | --- | --- | --- |
| Joint site | Prevalence of pain at site (%)  N= | Odds ratios for associations with pain at other anatomical sites^a^ | | | | | | | | | | | | | | | | | | | | | | | | |
|  |  | **Nk** | **Lw Bk** | **Up Bk** | **R**  **Sh** | **L**  **Sh** | **R**  **Elb** | **L**  **Elb** | **R**  **Wr** | **L**  **Wr** | **R**  **Hd** | **L**  **Hd** | **R**  **Th** | **L**  **Th** | **R**  **Hip** | **L**  **Hip** | **R**  **Gr** | **L**  **Gr** | **R**  **Kn** | **L**  **Kn** | **R**  **Ak** | **L**  **Ak** | **R**  **Ft** | **L**  **Ft** | **R**  **Blf** | **L**  **Blf** |
| **Nk** | 40.3 |  |  |  |  |  |  |  |  |  |  |  |  |  |  |  |  |  |  |  |  |  |  |  |  |  |
| **LwBk** | 62.1 | **2.9** |  |  |  |  |  |  |  |  |  |  |  |  |  |  |  |  |  |  |  |  |  |  |  |  |
| **UpBk** | 14.4 | **8.1** | **2.6** |  |  |  |  |  |  |  |  |  |  |  |  |  |  |  |  |  |  |  |  |  |  |  |
| **R Sh** | 31.3 | **2.2** | 1.4 | 1.6 |  |  |  |  |  |  |  |  |  |  |  |  |  |  |  |  |  |  |  |  |  |  |
| **L Sh** | 35.3 | 1.4 | 1.4 | 1.6 | **5.3** |  |  |  |  |  |  |  |  |  |  |  |  |  |  |  |  |  |  |  |  |  |
| **R Elb** | 10.5 | **3.1** | 1.4 | 1.5 | 1.7 | 1.6 |  |  |  |  |  |  |  |  |  |  |  |  |  |  |  |  |  |  |  |  |
| **L Elb** | 10.5 | **5** | 0.8 | 1.8 | 1.7 | **4.7** | **36** |  |  |  |  |  |  |  |  |  |  |  |  |  |  |  |  |  |  |  |
| **R Wr** | 12.4 | 1 | 1.3 | 1.1 | 1.2 | 0.9 | **2.4** | 1.4 |  |  |  |  |  |  |  |  |  |  |  |  |  |  |  |  |  |  |
| **L Wr** | 13.4 | 1.3 | 1.4 | 1.3 | 1.7 | 1.5 | **2.2** | **3.3** | **95.2** |  |  |  |  |  |  |  |  |  |  |  |  |  |  |  |  |  |
| **R Hd** | 41.3 | 1.4 | 0.7 | 1.1 | 0.9 | 0.9 | 0.8 | 0.8 | **2.3** | 1.5 |  |  |  |  |  |  |  |  |  |  |  |  |  |  |  |  |
| **L Hd** | 39.8 | 1.2 | 0.7 | 1.1 | 1.1 | 1 | 0.9 | 0.9 | **2.5** | 1.7 | **706** |  |  |  |  |  |  |  |  |  |  |  |  |  |  |  |
| **R Th** | 31.8 | 1.7 | 1 | 1.8 | 0.9 | 0.9 | 1.5 | 1 | 1.8 | 1.1 | **2.1** | **2.1** |  |  |  |  |  |  |  |  |  |  |  |  |  |  |
| **L Th** | 31.8 | 1.4 | 0.8 | 1 | 0.9 | 0.9 | 1.8 | 1.2 | **2.1** | 1.3 | 1.6 | 1.6 | **56.2** |  |  |  |  |  |  |  |  |  |  |  |  |  |
| **R Hip** | 33.3 | 1.4 | 1.5 | 1.2 | 2 | **2.5** | 1.3 | 1.4 | 1.3 | 1.7 | 0.8 | 0.6 | 0.5 | 0.6 |  |  |  |  |  |  |  |  |  |  |  |  |
| **L Hip** | 31.3 | 1.9 | 1.2 | 1.5 | **2.4** | **2.5** | 1.7 | 1.5 | 1.8 | 1.7 | 1.2 | 0.9 | 0.8 | 1.1 | **21** |  |  |  |  |  |  |  |  |  |  |  |
| **R Gr** | 10.5 | **2.8** | **4** | 1 | 1.7 | 1.4 | 1.3 | **2.1** | 0.7 | 1.7 | 0.7 | 0.8 | 0.3 | 0.3 | **4.3** | **2.8** |  |  |  |  |  |  |  |  |  |  |
| **L Gr** | 9.0 | **10** | **3.2** | 1.8 | 1.8 | 1.9 | **2.4** | 1.6 | 1.4 | **2.1** | 1.7 | 1.9 | 0.7 | 0.7 | 1.8 | **3.2** | **38** |  |  |  |  |  |  |  |  |  |
| **R Kn** | 73.6 | 1.2 | 1.5 | 0.9 | 1.6 | 1 | **3.5** | 1.4 | 1.3 | 1 | 0.9 | 0.7 | 0.9 | 0.8 | 1.7 | 0.7 | 0.9 | 1.2 |  |  |  |  |  |  |  |  |
| **L Kn** | 66.2 | 1.4 | 1.6 | 0.8 | 0.9 | 1.2 | **3.3** | 1.5 | 1.5 | 1.6 | 0.9 | 0.9 | 1 | 1.1 | 1.2 | 1.5 | 1.3 | **4.5** | **5.3** |  |  |  |  |  |  |  |
| **R Ak** | 9.5 | 0.7 | 0.5 | 1.1 | 0.3 | 0.8 | 1 | 1.4 | 1 | 1.2 | 1.2 | 0.6 | 0.6 | 0.9 | 0.9 | 1 | 0.9 | 1.2 | 1.5 | 0.6 |  |  |  |  |  |  |
| **L Ak** | 8.0 | 0.5 | 0.4 | 0.8 | 0.4 | 0.8 | 1.1 | 1 | 1.7 | 1.1 | **2.3** | 1.8 | 1.2 | 1.1 | 0.5 | 1.5 | 0.5 | 0.6 | 0.8 | **2.4** | **22** |  |  |  |  |  |
| **R Ft** | 8.5 | 0.8 | 0.6 | 0.3 | 1.2 | 0.7 | 1 | 1 | 1 | 1 | 1.8 | 1.1 | **2.4** | 1.7 | 1.2 | 1.6 | 1 | 1 | 0.8 | 0.9 | 1.3 | **2.7** |  |  |  |  |
| **L Ft** | 9.0 | 0.7 | 1 | 0.3 | 0.6 | 0.9 | 1 | 1 | 0.3 | 1 | 0.9 | 0.4 | 1.6 | 1.1 | 1.9 | 1.3 | 1 | 1 | 1.2 | 1.3 | 1.4 | **4.4** | **36** |  |  |  |
| **R Blf** | 18.9 | 0.8 | 0.5 | 0.9 | 1.1 | 1 | 1.2 | **3** | **3** | **2.5** | **4.4** | **3.6** | 1.8 | **2.8** | 0.9 | 1.1 | 0.7 | 0.9 | 0.9 | 0.7 | **2.4** | **3.3** | 1.4 | 0.9 |  |  |
| **L Blf** | 15.0 | 0.9 | 1 | 1.2 | 0.8 | 1.1 | 1.8 | 2 | **2.4** | **3.3** | **4** | **4.1** | 1.9 | **2.9** | 0.7 | 0.8 | 1 | 0.7 | 0.7 | 0.9 | **2.2** | **2.2** | 0.3 | 0.8 | **36** |  |
| Values are odds ratios (odds of pain in joint in column given pain in joint site shown in column for example odds ratio of 2.9 for having lower back pain if individual has neck pain)  ^a^ Odds ratios are adjusted for sex and age. **Bold** indicates a significant association.  R=right, L=left, Nk=neck, LwBk=lower back, UpBk=upper back, Sh=shoulder, Elb=elbow, Wr=wrist, Hd=hand, Th=thumb, Gr=groin, Kn=knee, Ak=ankle, Ft=foot, Blf=ball of foot | | | | | | | | | | | | | | | | | | | | | | | | | | |
